# Extracurricular physical activities and academic achievement in Saudi female physical education students: the mediating effect of motivation, enjoyment, and BMI

**DOI:** 10.3389/fpsyg.2025.1420286

**Published:** 2025-03-26

**Authors:** Mohamed Frikha

**Affiliations:** Department of Physical Education, College of Education, King Faisal University, Al-Ahsa, Saudi Arabia

**Keywords:** extra-class physical activity, psychological needs satisfaction, intrinsic and extrinsic motivation, academic performance, enjoyment, mediation analysis

## Abstract

**Background:**

Consistent with the self-determination theory and the trans-contextual model of autonomous motivation in education, the present investigation aimed to analyze the relationship between extracurricular physical activity (ECPA) and academic achievement (AA), and the mediating effects of enjoyment, motivation, and BMI among Saudi female physical education (PE) students.

**Materials and methods:**

The study followed as a descriptive cross-sectional design where a sample of 471 female PE students completed a self-administered questionnaire involving questions about their sociodemographic background, extracurricular physical activity practice, school performance, and the PE motivation and enjoyment questionnaires. Responses were collected between December 2022 and January 2023. Path analyses were chosen as a statistical method to understand the associations between the variables.

**Results:**

The theory-based model showed an acceptable fit with the data: (i) Standardized Root Mean Square Residual, *SRMR* = 0.084; (ii) comparative fit index, *CFI* = 0.952; (iii) Non-normed Fit Index, *NNFI* = 0.956; and (iv) root mean square error approximation, *RMSEA* = 0.051 (with CI 90%: 0.048-0.072). The Analysis revealed no significant direct effect of ECPA on AA (*ß* = 0.052, *p* > 0.05), and a significant indirect effect of enjoyment, intrinsic motivation, and BMI accounted for 20.04% of the total effect (22.9%).

**Conclusion:**

The enjoyment, intrinsic motivation, and BMI mediate the relationship between ECPA and AA in female PE students. The findings carry implications for PE teachers, principals and program makers in encouraging ECPA, supporting intrinsic motivation, enjoyment, and body image perception in Saudi female PE students.

## 1 Introduction

The benefits of regular physical activity (PA) throughout the life span were previously established by a myriad of studies. Recent studies have provided scientific evidence of this occurrence, and highlighted the importance of regular PA throughout the lifespan (Ungvari et al., 2023; Radak et al., 2019). According to Mahindru et al. (2023) and Brymer and Davids (2016), PA reduces the risk of disease, stress, mental fatigue, and aggressive behavior. It improves physical and life skills; psychological and emotional well-being; and positive mood and concentration. According to World Health Organization recommendations, adults should engage in 150–300 min of moderate-intensity PA, 75–150 min of vigorous-intensity PA, or some equivalent combination of moderate-intensity and vigorous-intensity aerobic PA per week (Bull et al., 2020).

### 1.1 Physical activity, BMI, and academic achievement

PA and body mass index (BMI) are considered to be key elements affecting academic performance in university students. Nonetheless, the relationships between PA, BMI and AA are still under debate for missing firm conclusions in the topic. In a systematic review, Barbosa et al. (2020) identified either minor positive or varied correlations between physical activity and academic performance. Their meta-analytical study indicated that the impact of physical activity on academic achievement was generally negligible, with some evidence suggesting small to moderate positive effects. Moreover, frequency of PA practice seems to have an important effect on AA. Indeed, Hossen et al. (2020) found that university students who engaged in sports or physical exercise on a near-daily basis, or approximately four to five times per week, exhibited superior health outcomes; however, they tended to achieve lower academic performance compared to those who participated in such activities for only 2–3 days each week. Nonetheless, a greater physical fitness level (i.e., high VO2 max rates) was found to be positively related to greater academic achievement (Redondo-Flórez et al., 2022). In the Saudi context, the academic achievement was found to be significantly negatively correlated with BMI in female university students (Alhazmi et al., 2021), but not correlated with regular PA practice (Frikha et al., 2022). Likewise, female students with a normal BMI had a significantly greater grade point average (GPA); however, a low GPA was significantly correlated with a high BMI (Alhazmi et al., 2021). Besides, it has been suggested that BMI was significantly negatively correlated with student’s final grades (Anderson and Good, 2017), and that high BMI values may also be associated with sedentary behaviors, and body image dissatisfaction in female medical students (Mohamed and Idrees, 2023). For many female students suffering from high BMI and negative body image and dissatisfaction, sports and PA can be intimidating, especially if they perceive themselves as lacking motor skills or athletic abilities (Engels and Freund, 2019; Cowley et al., 2021).

### 1.2 Physical activity, enjoyment and motivation perceptions

On the other hand, regular PA can be sustained most effectively if it is perceived as enjoyable (Rodrigues et al., 2021; Engels and Freund, 2019). When students find enjoyment in participating in PA, they are more likely to engage actively, develop a positive attitude, and continue pursuing an active lifestyle beyond the classroom (Lohbeck et al., 2019). Students’ enjoyment of PE is examined from three distinct dimensions: pleasure, flow, and recovery (Toselli et al., 2022), where pleasure is defined as an anticipation of physical education, flow is defined as a positive feeling during physical education, and recovery is defined as an expected result. One of the key benefits of emphasizing enjoyment in PE is the reduction of barriers to participation. For many female students suffering from negative body image and dissatisfaction, sports and PA can be intimidating, especially if they perceive themselves as lacking natural athletic abilities (Engels and Freund, 2019; Cowley et al., 2021). In a recent descriptive cross-sectional study Frikha et al. (2024b) highlighted the effect of PAE on basic psychological needs, motivation and academic integration in female PE university students. Beside, in a review, Kerner et al. (2022) highlighted that body dissatisfaction in PE impairs adolescents’ satisfaction and enjoyment of PE, which are considered key characteristics of intrinsic motivation. Therefore, by creating an environment that prioritizes enjoyment and motivation, teachers can help students overcome these barriers and develop a sense of confidence and self-efficacy (Fu et al., 2023; De Souza-Pajuelo et al., 2021; Jankauskiene et al., 2022).

Studies have shown that students participate in more extracurricular physical activities (ECPAs) if they enjoy PE classes (Chu et al., 2017; Sanchez-Oliva et al., 2014) or if they have high emotional and motivational experiences in PE classes (Fierro-Suero et al., 2022). According the self-determination theory (SDT) (Ryan and Deci, 2017), there are four qualitatively different forms of motivational regulation along a continuum of self-determination ranging from intrinsic motivation to external regulation. Satisfying the basic psychological needs within a given activity is a prerequisite for developing autonomous motivation, which is represented by intrinsic motivation and identified regulation. In contrast, frustration of such psychological needs is predicted to lead to controlled motivation, which is comprised of introjected and external regulations. In a study conducted by Fierro-Suero et al. (2023), it was demonstrated that the autonomous motivation became a strong predictor of students’ intentions to participate in physical activities, whereas academic achievement was determined by both autonomous motivation and emotional factors. Kalajas-Tilga et al. (2020), concluded that enhancing moderate-to-vigorous physical activity (MVPA) in adolescents requires the increase of their intrinsic motivation toward PE. Likewise, recent studies (Llanos-Muñoz et al., 2023; Machunde and Ilomo, 2022) highlighted that the more students participate in extracurricular activities, the more their learning motivation increases. Thus, to optimally promote student interest in PE, it is crucial to identify the factors that influence students’ sense of enjoyment and motivation perceptions.

### 1.3 Extracurricular physical activities and academic achievement

Physical education sessions in school and university contexts are ideal environments for promoting physical activity (Mounesan et al., 2012) and reducing sedentary behaviors (Mayorga-Vega et al., 2018). Conversely, recent findings highlighted the low demand for PA offered in schools (do Nascimento et al., 2019). Indeed, students in PE sessions were shown to spend nearly 65% of their time without engaging in intense PA (da Costa et al., 2017). Consequently, the need for extracurricular PA (ECPA) appears to be highly requested in stimulating PE objectives (do Nascimento et al., 2019).

Defined as activities that are generally optional and that take place outside the classroom, ECPA can include a wide range of options from organized sports to non-organized PA. ECPA were shown to promote the development of personality and interpersonal skills (Ivaniushina and Zapletina, 2015). They are vital in the socialization of children and adolescents, and they provide a valuable resource for personal development and the acquisition of social competencies (Ivaniushina and Zapletina, 2015). According the trans-contextual model of Hagger and Chatzisarantis (2016), motivation plays a key component of the relation between in-class and outside of school PA engagement. It (the trans-contextual model) elucidates the mechanisms through which autonomous motivation for activities within a PE setting can forecast autonomous motivation for PA beyond the school environment, as well as the actual participation in PA outside of school (Hagger and Chatzisarantis, 2016).

Moreover, the scientific literature shows that students can attain greater academic achievement by participating in planned ECPA. For instance, engagement in ECPA aids in the development of many talents and dispositions that aid in academic success. These include cognitive abilities that go beyond what is taught (Real-Pérez et al., 2022) as well as non-cognitive attitudes and skills such as self-esteem (Lee et al., 2018). Moreover, previous studies have demonstrated that ECPA help improving interactions with peers and, as a result, provide information, which can aid in academic achievements (Sabirova and Zinoviev, 2016). This interaction with adults and peers affects students’ goals and decisions, which are often more school-focused (Fujiyama et al., 2021).

Extracurricular activities allow students to explore their particular abilities and to promote skills and interests (Buckley and Lee, 2021). Hence, extracurricular participation might be expected to boost academic achievement through the collection of information and skills (Real-Pérez et al., 2022; Kravchenko and Nygård, 2022). Recent research has shown physical (Greco et al., 2019), cognitive (Real-Pérez et al., 2022), social (Kravchenko and Nygård, 2022), and health (Ricci et al., 2020) benefits of ECPA. Furthermore, there is growing evidence that ECPA can have a significant impact on academic achievement through its effect on cognitive and motor abilities (Frikha and Alharbi, 2023; Real-Pérez et al., 2022).

### 1.4 Women’s PA practices in the Saudi context

Research on the Saudi population has shown that most Saudi teenagers do not perform the recommended 60 min of physical activity each day, and as a result, 28–30% of adolescents are overweight or obese (Alasqah et al., 2021). According to the Gulf Cooperation Council, Saudi Arabia has the second highest rate of obesity among women (Al-Hazzaa and AlMarzooqi, 2018). For instance, recent findings (AL-Shahrani, 2020) highlighted that, only 16% of Saudi women regularly practice PA, at a rate of three times a week. Nonetheless, the rapid changes occurring in Saudi society in relation to the 2030 Kingdom Reform Vision underscore the importance of promoting sport and PA in building social capital (Al Derham, 2023). In particular, women in Saudi Arabia were newly allowed to engage in public PA, and PE programs were recently introduced in schools and universities (since the 2018 academic year) (Aljehani et al., 2022). However, the educational institutions continue to offer exclusively non-mixed learning environment (AlJuhani, 2023), which find roots in the Arab-Islamic cultural norms and tradition. Beside, studies showed several barriers that still reduce women’s participation in PA, such as a lack of motivation (Al-Otaibi, 2013), limited family support (Lysa, 2020), gender segregation and sociocultural pressure (Aljehani et al., 2022). According to AL-Shahrani (2020) and Alghamdi and Aldossari (2022), Saudi women participate in sports for a variety of reasons, including fitness, agility, health preservation, and self-satisfaction. Hence, females with higher intrinsic and extrinsic motivation are likely to have a high level of pleasure and degree of self-determination (AL-Shahrani, 2020; Coroiu and Dintica, 2023).

### 1.5 The current study

The relationship between engaging in ECPA and achieving academic success has been extensively examined on a global scale. Nevertheless, the findings regarding this association have presented conflicting results, leading to ongoing debates and uncertainties regarding its actual influence (Suárez-Cano et al., 2023; Redondo-Flórez et al., 2022). Thus, studying the relationship between PA practices and academic achievement is still an interesting research topic (Suárez-Cano et al., 2023; de Greeff et al., 2018). Therefore, examining the relationship between ECPA and educational outcomes is also highly important (Coroiu and Dintica, 2023; Fujiyama et al., 2021) especially in the context of Saudi Arabia. Nonetheless, our understanding of the associations between ECPA, AA, PAE, motivation, and BMI in Saudi female PE students is still limited. To the best of our knowledge, no previous studies have examined the mediating effects of enjoyment, motivation, and BMI on the relationship between ECPA and AA. It seems that ECPA promotes AA, while PAE, motivation, and BMI mediate this relationship. Thus, analyzing and identifying the factors that enhance students’ AA with regard to ECPA, motivation, BMI and PAE remains crucial. Therefore, this study aimed to determine whether PAE, motivation, and BMI can mediate the relationship between PAE and AA among female physical education students.

Based on the Self-Determination Theory (SDT) and previous research, we put forward the following hypothesis:

*H1*: Students ECPA would positively predict their motivation, PAE and BMI.

*H2*: Motivation, PAE and BMI predict AA in female PE students.

H3: ECPA would increase AA through the mediation of PAE, motivation, and BMI.

## 2 Materials and methods

### 2.1 Research design and sample size calculation

A descriptive cross-sectional design was used in the present study. Based on geographical randomization, four public universities were chosen in the western, central, northern, and eastern regions of Saudi Arabia. The sample size (n) was estimated using Daniel (1999) method, assuming a prevalence (p) of 50%, a needed confidence level of 95%, a margin of error (e) of 5%, and an associated z score of 1.96. With a 20% dropout rate, a minimum of 357 students were asked to participate. Nonetheless, to increase student involvement, the Google Form Questionnaire link was forwarded to practically all regular PE female students at the selected institutions (1,300 requests) after contacting each university’s dean of information technology. The study’s ultimate sample size was determined by the 471 affirmative replies received. Responses were collected between December 2022 and January 2023.

### 2.2 Participants

Female PE students (age: 20.51 ± 1.92 years; BMI: 21.27 ± 2.91 kg/m2), regularly studying at Saudi universities (level to level 4 students from Taif University, King Saud University, Hail University, and Hafr Al-Batin University;) were asked to participate in the present study. The inclusion criteria were as follows: (i) Study regularity in a PE department or college. Students who studied in preparatory year programs or who had less than 75% attendance were not asked to participate in (ii) the presence of practical and theoretical courses on the student’s schedule or (iii) the completion of all questionnaire questions. All the respondents were properly aware of the study’s purpose. All participants were informed that their replies would be kept anonymous and that no information that could reveal their identities would be requested. By clicking a specific box at the beginning of the questionnaire, all participants provided written informed consent and agreed to have their comments published.

### 2.3 Questionnaires

In the present investigation, we used the items from previous questionnaires, namely, the PE motivation scales (PE-MS) of Sulz et al. (2016) to assess intrinsic and extrinsic motivation, and the questionnaire for the assessment of students’ enjoyment (pleasure, flow, and recovery) in physical education (QUAEPE) of Engels and Freund (2020). The study also collected demographic information (age, weight, height, weekly curricular PE practical sessions, weekly extracurricular moderate-to-vigorous PA practices, and grade point average).

#### 2.3.1 The PE motivation scale (PE-MS)

The PE-MS included nine items and assessed intrinsic motivation (items 1, 4, and 7), extrinsic motivation (items 2, 5, and 8), and amotivation (items 3, 6, and 9). A 5-point Likert scale was used (1 = strongly disagree, 2 = disagree, 3 = neutral, 4 = agree, 5 = strongly agree) in the PE-MS. Values of intrinsic and extrinsic motivations were retained for analysis. A higher total score implies a greater degree of motivation. The reliability of the Arabic version of the PE-MS was previously verified (Frikha et al., 2024a) with satisfactory Cronbach’s alpha values (0.823 for intrinsic motivation, 0.836 for extrinsic motivation, and 0.849 for amotivation).

#### 2.3.2 The questionnaire for the assessment of students’ enjoyment in physical education (QUAEPE)

The students’ enjoyment in the physical education questionnaire (QUAEPE) of Engels and Freund (2020) involved nine items: pleasure (items 10, 11, 12), flow (items 13, 14, 15), and recovery (items 16, 17, 18). A 4-point Likert scale (0 = never, 1 = sometimes, 2 = often, 3 = always) was used. A higher total score implies a greater degree of enjoyment. The reliability of the Arabic version of the QUAEPE was verified with satisfactory Cronbach’s alpha values (0.806 for pleasure, 0.795 for flow, and 0.792 for recovery).

#### 2.3.3 Validity and reliability of the Arabic versions of the QUAEPE

The QUAEPE was translated into Arabic and adapted to the Saudi context by two bilingual translators. The translated versions were then back retranslated into the English language by three bilingual experts. A revision according to the recommendations of Beaton et al. (2000) was conducted, and the necessary adjustments were made. The validity and reliability of the obtained Arabic version of the QUAEPE was tested, through a pilot study conducted with a sample of PE students (n = 31), according Bujang et al. (2024) recommendations, using exploratory factor analysis (EFA) and Cronbach’s alpha for factorial validity and internal reliability, respectively. The validity and reliability of the QUAEPE were verified with EFA using all participants’ responses.

### 2.4 Data analysis

Extracurricular PA (ECPA) was assumed to be the independent variable X, and academic achievement (AA) was the dependent variable Y. Enjoyment perception, intrinsic and extrinsic motivation, and BMI were assumed to be intermediary variables (M1, M2, M3, and M4, respectively). ECPA thus positively affects AA, and intrinsic, extrinsic motivation and BMI play intermediary roles in the relationship between ECPA and AA. The statistical analysis was performed using SPSS V.26 (IBM, Armonk, NY, USA) and SPSS AMOS (Version 23.0; IBM Corp.). We used Pearson correlation and regression analyses to evaluate the associations between indicators. The Hayes process macro (Model 4) was used to analyze the direct and indirect impacts of ECPA on AA. The total effect is the sum of the direct and the indirect effects. The goodness fit of the model was verified using Standardized Root Mean Square Residual (SRMSR), comparative fit index (CFI), Non-normed Fit Index (NNFI), and root mean square error approximation, (RMSEA, with CI 90%) (Schermelleh-Engel et al., 2003; Hu and Bentler, 1998). Exploratory factor analysis (EFA) using principal component analysis and Cronbach’s alpha were used to verify the validity and reliability of the questionnaire. Descriptive data are summarized as the means and standard deviations or proportions of the total population. Normality was tested using histograms and absolute values of skewness, and all values were < 2 (Kim, 2013). The significance level was set at *p* < 0.05.

## 3 Results

### 3.1 Validity and reliability of the Arabic versions of the QUAEPE

Exploratory factor analysis was performed using the principal component analysis extraction approach and varimax rotation. Using responses from the testing sample (N = 31), the results showed that Kaiser–Meyer–Olkin (KMO) sampling adequacy was above the acceptable threshold of 0.6 (KMO = 0.757); Bartlett’s sphericity test result was statistically significant (*p* < 0.001). Three components with eigenvalues >1 (3.851, 3.161, and 2.834) were identified and maintained (the cumulative variance was set at 84.279%).

Concerning the internal reliability of the QUAEPE, Cronbach’s alpha values were set at 0.902 (all items included), and 0.830, 0.899, and 0.855 for pleasure, flow, and recovery, respectively.

The validity and reliability were verified using the responses of all participants (N = 471). The results showed that Kaiser–Meyer–Olkin (KMO) sampling adequacy was above the acceptable threshold of 0.6 (KMO = 0.925); Bartlett’s sphericity test result was statistically significant (*p* < 0.001). Three components with eigenvalues >1 (3.203, 4.566, and 2.975) were identified and maintained (the cumulative variance was set at 85.668%).

Concerning the internal reliability of the QUAEPE, Cronbach’s alpha values were set at 0.874 (all items included), and 0.871, 0.865, and 0.932 for pleasure, flow, and recovery, respectively. Therefore, the Arabic versions of the QUAEPE scales was found to be reliable and valid for measuring enjoyment.

### 3.2 Sociodemographic characteristics

In total, 471 female PE students participated in the study, for a response rate of 39%. The majority of participants were novices in university life (86.2% from the first and second levels). Table 1 shows the descriptive statistics of the variables, age, body mass index, university, curricular and extracurricular PA, and GPA (Table 1).

**Table 1:** Sociodemographic characteristics of the respondents (*n* = 471).

Variables	Values *
Age	20.51 ± 1.92 years
Global body mass index (BMI)	21.27 ± 2.91 kg/m^2^
BMI frequency	
BMI < 18.5	77 (16.3%)
18.6 < BMI < 25	350 (74.3%)
25.1 < BMI < 30	40 (8.5%)
BMI > 30.1	4 (0.8%)
Universities	
Taif University (TU)	209 (44.4%)
King Saud University (KSU)	96 (20.4%)
Hail University (HU)	69 (14.6%)
University of Hafr Al-Batin (HBU)	97 (20.6%)
PE study level	
First and second year	404 (85.8%)
Third and fourth year	67 (14.2%)
Grade point average (GPA)	
1 < GPA ≤ 2	24 (5.1%)
2 < GPA ≤ 3	106 (22.5%)
3 < GPA ≤ 4	150 (31.8%)
4 < GPA ≤ 5	191 (40.6%)
Curricular PA/week	
Two or less	238 (50.5%)
Between three and four	199 (42.3%)
Five or more	34 (7.2%)
Extracurricular PA/week	
No PA	119 (25.3%)
Twice a week	158 (33.5%)
Three times a week	160 (34.0%)
Daily	34 (7.2%)

* Values are given as n (%) unless otherwise stated.

### 3.3 Relationships between extracurricular PA, motivation, enjoyment, and BMI

The results showed that there were significant correlations between ECPA and AA (*r* = 0.281, *p* < 0.001), between ECPA and all other mediators (enjoyment, *r* = 0.432, *p* < 0.001; IM, *r* = 0.277, *p* < 0.001; EM, *r* = 0.234, *p* < 0.001; and BMI, *r* = −0.138, *p* < 0.001). Significant correlations were observed between AA and all the mediators (enjoyment, *r* = 0.454, *p* < 0.001; IM, *r* = 0. 0.528, *p* < 0.001; EM, *r* = 0.320, *p* < 0.001; and BMI, *r* = −0.293, *p* < 0.001). The correlations between the study variables allowed a mediation model to be constructed to explore the mechanism of all the mediators’ effects on the relationship between ECPA and AA (Table 2).

**Table 2:** Correlations between extracurricular PA, motivation, enjoyment, body mass index and grade point average in female students (*n* = 471).

		ECPA	GPA	BMI	IM	EM	PAE
ECPA	Pears Correl	1					
	95% CI						
GPA	Pears Correl	0.281**	1				
	95% CI	0.181 –0. 314					
BMI	Pears Correl	−0.138**	−0.293**	1			
	95% CI	–0.112 – 0.243	−0.199 – –0.543				
IM	Pears Correl	0.277**	0.528**	−0.125**	1		
	95% CI	0.223–0.411	0.484–0.629	–0.111 – 0.240			
EM	Pears Correl	0.234**	0.320**	−0.121*	0.454**	1	
	95% CI	0.203–0.316	0.320–0.472	–0.101 – 0.270	0.442–0.592		
PAE	Pears Correl	0.432**	0.454**	−0.221**	0.451**	0.344**	1
	95% CI	0.466–0.613	0.411–0.591	−0.167 – –0.344	0.552–0.690	0.312–0.551	

ECPA: extracurricular PA; GPA: grade point average; BMI: body mass index; IM: intrinsic motivation; EM: extrinsic motivation; PAE: PA enjoyment; CI: confidence interval. Correlations are significant at: * 0.05; ** 0.01 levels (2-tailed).

### 3.4 Mediation analysis

The study assessed the mediating role of enjoyment, motivation (intrinsic and extrinsic), and BMI on the relationship between ECPA and AA in female physical education students. The results revealed significant indirect effects of ECPA on AA (complementary partial mediations) through enjoyment (*ß* = 0.088, t = 4.556, p < 0.001), IM (*ß* = 0.105, *t* = 8.578, *p* < 0.001), and a competitive partial mediation of BMI (*ß* = 0.026, *t* = −5.006, *p* < 0.001). No significant indirect effect of EM was recorded, with confidence interval (CI 95%) including zero between the lower and upper limits bootstraps (*ß* = 0.011, *t* = 1.029, *p* > 0.05). Furthermore, the direct effect of ECPA on AA in the presence of the mediators was not significant, with confidence interval (CI 95%) including zero between the lower and upper limits bootstraps (*ß* = 0.052, *t* = 1.261, *p* > 0.05). Hence, all the mediators, enjoyment, intrinsic motivation, and BMI, partially mediate the relationship between ECPA and AA. The mediation summary is presented in Table 3.

**Table 3:** Mediation analysis summary.

Total effect ECPA→AA	Direct effect ECPA → AA	Relationship	Indirect effect	Boot SE	95% CI		*t*		Decision
					LB	UB			
0.229 (0.000)	0.052 (0.41)	ECPA→PAE → AA	0.088	0.021	0.05	0.131	4.556	a_1_ b_1_ c´ > 0	Complementary PM
		ECPA→IM → AA	0.105	0.02	0.067	0.148	8.578	a_2_ b_2_ c´ > 0	Complementary PM
		ECPA→EM → AA	0.011	0.009	−0.060	0.029	1.029	a_3_ b_3_ c´ > 0	NS
		ECPA→BMI → AA	0.024	0.010	0.006	0.045	−5.006	a_4_ b_4_ c´ < 0	Competitive PM

ECPA: extracurricular physical activities; AA: academic achievement; PAE: physical activity enjoyment; IM: intrinsic motivation; EM: extrinsic motivation; BMI: body mass index; LB: lower band; UB: upper band; PM: partial mediation; NS: non-significant.

**Figure 1:**
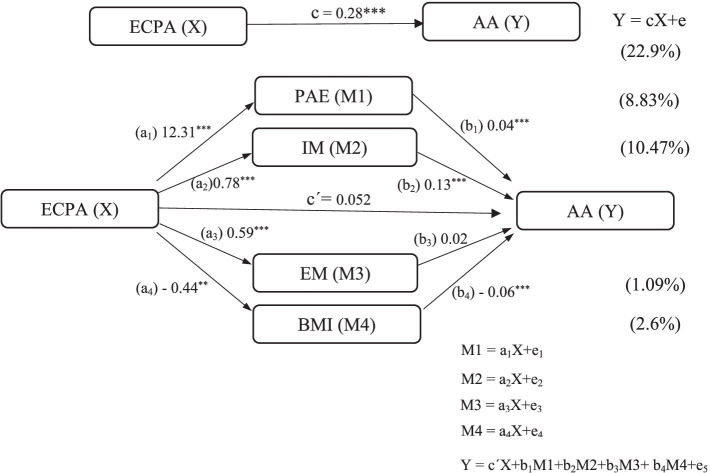
Mediation model diagram and significance. ECPA: extracurricular physical activity; PAE: physical activity enjoyment; IM: intrinsic motivation; EM: extrinsic motivation; BMI: body mass index; AA: academic achievement. ** Significant at *p* < 0.01; *** significant at *p* < 0.001.

The goodness fit of the model using SPSS AMOS revealed: (i) Standardized Root Mean Square Residual, SRMR = 0.084; (ii) comparative fit index, CFI = 0.952; (iii) Non-normed Fit Index, NNFI = 0.956; and (iv) root mean square error approximation, RMSEA = 0.051 (with CI 90%: 0.048–0.072). The coefficients c, a1, b1, a2, b2, a4, and b4 were all significant, indicating that PAE, IM, and BMI played partial intermediary roles. Bootstrap methods were used to repeat the sampling process 5,000 times to test the mediating effect. All 95% confidence intervals of the mediation effects of paths do not include 0, which indicates that the mediation effects are statistically significant and account for 24.3% of the effect. In summary, the results showed that ECPA did not directly affect AA. While AA is impacted by PAE, IM, and BMI (Figure 1).

## 4 Discussion

The aim of the present investigation was to assess the relationships between extracurricular physical activity (ECPA) and academic achievement (AA), as well as the mediating effects of motivations (intrinsic and extrinsic; IM, EM), physical activity enjoyment (PAE), and BMI, on female Saudi PE students. Path analysis was used, and the findings revealed the following: (i) IM, EM, PAE and BMI are predicted by ECPA (H1); (ii) IM, PAE and BMI are predictors of AA; (H2); (iii) the model proposed presented an acceptable goodness fit: there are complementary partial mediation effects of PAE, IM, and competitive minor contribution of BMI in the relationship between ECPA and AA, but not by EM; there was no direct effect of ECPA on AA(H3).

The study sample included Saudi female PE students being part of a society witnessing fundamental changes and transitions toward the Kingdom 2030 Reform Vision, in which the focus is on both physical fitness and women’s leadership (Al Derham, 2023). Indeed, the results showed that 85.8% of the participants were students in level 1 and 2. Likewise, 74.7% practiced ECPA for at least two times per week in addition to the regular PE sessions in university. Hence, even though the majority of participants were novice students, with limited experiences in PE and in PA, we can assume that they are physically active (Bull et al., 2020; Aljehani et al., 2022; Lysa, 2020).

The present study showed positive relationships between ECPA, GPA, PAE, IM and EM in female PE students, which is in accordance with the findings of Frikha et al. (2024b) and with the finding of Guimaraest (2015), proving evidence that engagement in sports and physical activities can facilitate significant developmental transformations in the self-perceptions, self-esteem, and academic performance in female university students. Moreover, the positive relationship between students’ enjoyment in PE classes and their engagement in extracurricular PA was previously highlighted in several studies (Chu et al., 2017; Sanchez-Oliva et al., 2014). According the SDT (Ryan and Deci, 2017), the intrinsic motivation is the most self-determined, or autonomous form of motivation. Thus, when a person is intrinsically motivated, he or she willfully engages in action because it is naturally intriguing or enjoyable (Weman-Josefsson et al., 2015).

Besides, according Obergriesser and Stoeger (2020), and Fierro-Suero et al. (2023), emotions, and specifically enjoyment, strongly affect motivation and self-regulation in students, which in turn have a predictive impact on actions that support and sustain AA. In this context, findings have shown that teacher autonomy support is a predictor of intrinsic motivation and perceived competence, and that competence is still the unique predictor of AA in university students (Jeno et al., 2023).

In their study, Pekrun et al. (2011) noted that enjoyment is positively correlated with intrinsic motivation. Students who are intrinsically motivated and have high competence and relatedness perceptions are more likely to have a high sense of enjoyment, which eventually leads to greater academic accomplishment (León et al., 2015). Furthermore, according to Aljehani et al. (2022), Saudi women’s participation in physical activity can be explained by two main factors: one is the personal motivation stemming from an internal desire for physical activity and a healthy lifestyle (intrinsic motivation), and the second is the family support (extrinsic motivation), which includes encouragement and praise. However, the present findings are still at odds with other showing no relationship between psychological needs satisfaction (autonomy, competence, and relatedness), GPA, and PA in a sample of male and female university students (Frikha et al., 2022). Even though both studies were conducted in the same social context, and with similar participants’ characteristics (i.e., Saudi PE students), the discrepancies in the findings may be related to differences in the learning modalities (online learning during the COVID-19 pandemic restriction vs. face-to-face learning modality in the present study) or to the participants’ gender characteristics (male and female vs. female only in the present study).

To the best of our knowledge, this is the first study examining the mediating effect of PAE, motivation, and BMI on the relationship between ECPA and AA in Saudi female PE students. The theory based model showed an acceptable goodness fit according the recommendations of Schermelleh-Engel et al. (2003). The research findings suggest that female PE students not only need to be intrinsically motivated to have high AA, but also need to perceive a sense of enjoyment and to pay attention to BMI values.

The mediating effects of enjoyment, intrinsic motivation, and BMI demonstrated their importance in the relationship between ECPA and AA. Contrary to our expectations, the present study failed to establish a direct relationship between ECPA and AA or a mediating effect of EM, which demonstrated that their relationship was exclusive through PAE, IM, and BMI. These findings are in accordance with previous studies showing minor positive correlations between ECPA and AA (Barbosa et al., 2020; Balaguer et al., 2020), and with other studies mentioning that IM, autonomy and competence are predictors of AA, but not EM (Frikha et al., 2024a; Fierro-Suero et al., 2023). Nonetheless, it is still at odds with others showing a positive relationship between PA and AA (Redondo-Flórez et al., 2022; Alhazmi et al., 2021). These discrepancies could be related to the characteristics of the study participants. Indeed, Balaguer et al. (2020) stated that the effect of extracurricular activities may differ on students’ academic achievement depending on their age, sex or parental educational level. Moreover, in a review, Tanner (2017) highlighted that even engaging in extracurricular activities can potentially have adverse consequences when they result in an overload of personal schedules and lead students to primarily identify themselves based on their involvement in these activities rather than their academic pursuits. In addition, the non-significant mediating effect of EM demonstrated that it had no effect on the relationship between ECPA and AA. A similar conclusion was mentioned in Frikha et al. (2024a) and Bin Abdulrahman et al. (2023), where no effect of EM was found on GPAs of Saudi PE and medical students’, respectively. Likewise, the findings of the current study align with the work of Kamberi and Danuza (2022), indicating no significant relationship between AA and either introjected or identified forms of EM. Despite its role in driving effort and improving performance through the use of rewards and positive reinforcement strategies (Weman-Josefsson et al., 2015), EM seems to be not related with AA. The recent reforms in Saudi Arabia, particularly the Vision 2030 initiative, serve as a significant extrinsic motivator for women (Al Derham, 2023). Nevertheless, it remains plausible that these changes have not sufficiently transformed cultural norms or provided adequate social support, given that Saudi society continues to be characterized by deep-rooted religious, conservative, traditional, and family-oriented values (Al Derham, 2023).

Concerning the mediating effect of PAE, the present study findings are in accordance with those of Yan et al. (2023), revealing that enjoyment mediates the relationship between physical literacy and moderate to vigorous PA among college students, and with those of Engels and Freund (2020), indicating that social relatedness and perceived competence in PE classes partially mediate the effect of cooperative games on enjoyment. In addition, developing interest in and enjoyment of PA for life could be assured by identifying and implementing strategies to enhance motivation in PE by providing students with unstructured PA opportunities, allowing changes in their perceptions of the value of PA and leading to increased enjoyment in PE sessions (Kinder et al., 2020).

The present study findings showed that PAE and IM mediate the relationship between ECPA and AA, with a mediating effects of PAE and IM accounted at 8.83 and 10.47%, respectively, which suggests that their importance is well supported. However, the mediating effect of BMI (2.6%) is still minor and with limited importance in the relationship. This result suggest that BMI is not a strong predictor as PAE and IM for AA. Explanation could be found in the academic curricula in PE programs that encompass a variety of both theoretical and practical disciplines, where even students with elevated BMI could achieve high GPA. Moreover, and given the negative correlation between ECPA and BMI, and the reverse relationship between BMI and body image satisfaction (Mohamed and Idrees, 2023), we can assume that students non participating in ECPA are most likely those having higher BMI and then suffering from body dissatisfaction. Hence, ECPA affect AA through the mediation of students’ perceptions of body image satisfaction, which was previously demonstrated to affect both enjoyment and intrinsic motivation (Kerner et al., 2022). According to the observations made by Cowley et al. (2021) and Engels and Freund (2019), having negative body image, body dissatisfaction and high BMI, may leads female students to avoid engaging in PA.

Overall, these findings indicate that ECPA can play an important role in enhancing academic achievement if PAE, IM, and BMI are improved in PE female students. This means that even if female PE students actively engage in ECPA, the impact on their AA may not be reached, as they are not intrinsically motivated or not perceiving enjoyment in PE sessions, which is in accordance with the trans-contextual model of Hagger and Chatzisarantis (2016).

### 4.1 Study strengths, limitations and perspectives

This study represents an initial investigation into the mediating effect of PAE, motivation, and BMI on the association between ECPA and AA among female PE students in Saudi Arabia. Apprehending these relationships remains a crucial step in improving the academic performance of PE students. Nonetheless, some limitations merit discussion. First, the proportion of non-practicing extracurricular PAs in the present investigation is still relatively important (25.4% of participants), which may have influenced the results of the study. Second, since the present study was implemented in a sample of students with different academic experience (85.8% from the first and second years and 14.2% from the third and fourth years of study), with a majority of PE students considered as novice, thus any generalization of the results should be made with precaution. Third, we did not consider the type of extracurricular PA performed in the present study. For instance, as mentioned by several studies (Redondo-Flórez et al., 2022; Ivaniushina and Zapletina, 2015), each form of activity may be distinguished by its own distinct profile and could subsequently affect skills and competencies differently. The complexity of motivation becomes evident when considering its connection to students’ behavior, attitude, and learning context. This highlights the fact that employing various techniques alone may not yield the desired results, necessitating a more comprehensive examination of the individual, collective, and contextual factors involved (Assor et al., 2009). The autonomy support from PE teachers, peers and parents may affect autonomous motivation toward physical activities in PE and then the desire to participate to ECPA (Kalajas-Tilga et al., 2022). Considering the above highlighted limitations, further investigations are needed to elucidate the students’ behaviors according to goal orientation theory (Elliot, 2007), in a sample of PE students with diverse genders, academic and social backgrounds, to better enhance the external validity of the model. In addition, it will be interesting to investigate the mediating role of basic psychological needs in the relationship between PA in the PE context and PA outside of university according to the trans-contextual model of Hagger and Chatzisarantis (2016).

## 5 Conclusion

This study expands on previous research in physical education by exploring the different pathways that connect ECPA to AA. It discusses the influence of PAE, motivation (IM and EM), and BMI on this relationship. PAE, IM, and BMI are predicted by ECPA and are predictors of AA. The model explored in this study presents an acceptable goodness fit and provides a conceptual framework that clarifies the mediating effects of PAE, IM, and BMI on the relationship between ECPA and AA in Saudi female PE students. Considering the non-significant direct effect between ECPA and AA, Saudi female PE students may not have high AA, even with high levels of ECPA, if they are not intrinsically motivated, do not perceive a sense of enjoyment in PE sessions, or suffering from overweight resulting in body image dissatisfaction. Therefore, to promote AA in Saudi female PE students, teachers should encourage them to participate in ECPA, support their intrinsic motivation and enjoyment perceptions by giving meaning to things they do, and proposing pleasant PA. Beside, Saudi university principals and program makers should be more aware of the spread of the culture of extracurricular PA practice among female students.

## Data Availability

The raw data supporting the conclusions of this article will be made available by the authors, without undue reservation.
